# Prevalence of and risk factors for chlamydia in female outpatients with genital tract infections: a nationwide multi-center, cross-sectional study in China

**DOI:** 10.3389/fpubh.2023.1182108

**Published:** 2023-06-15

**Authors:** Ting Li, Zhaohui Liu, Dai Zhang, Qinping Liao, Shangrong Fan, Min Hao, Ying Hong, Xiufeng Huang, Huilan Wang, Zhengai Xiong, Hong Xu, Fengxia Xue, Min Xue, Xingsheng Yang, Jianqing Zhang

**Affiliations:** ^1^Department of Gynecology, Beijing Obstetrics and Gynecology Hospital, Beijing Maternal and Child Health Care Hospital, Capital Medical University, Beijing, China; ^2^Department of Obstetrics and Gynecology, Peking University First Hospital, Beijing, China; ^3^Department of Obstetrics and Gynecology, Beijing Tsinghua Changgung Hospital, School of Clinical Medicine, Tsinghua University, Beijing, China; ^4^Department of Obstetrics and Gynecology, Peking University Shenzhen Hospital, Shenzhen, Guangdong, China; ^5^Department of Obstetrics and Gynecology, Second Hospital of Shanxi Medical University, Taiyuan, China; ^6^Department of Obstetrics and Gynecology, Nanjing Drum Tower Hospital, Nanjing University Medical School, Nanjing, China; ^7^Department of Obstetrics and Gynecology, Women’s Hospital, School of Medicine, Zhejiang University, Hangzhou, China; ^8^Department of Obstetrics and Gynecology, The Second Hospital of Hebei Medical University, Shijiazhuang, China; ^9^Department of Obstetrics and Gynecology, The Second Affiliated Hospital of Chongqing Medical University, Chongqing, China; ^10^Department of Obstetrics and Gynecology, The First Affiliated Hospital of Guangxi Medical University, Nanning, China; ^11^Department of Obstetrics and Gynecology, Tianjin Medical University General Hospital, Tianjin, China; ^12^Department of Obstetrics and Gynecology, Third Xiangya Hospital of Central South University, Changsha, China; ^13^Department of Obstetrics and Gynecology, Qilu Hospital of Shandong University, Jinan, China; ^14^Department of Obstetrics and Gynecology, Qinghai Red Cross Hospital, Xining, China

**Keywords:** *Chlamydia trachomatis*, genital tract infection, risk factor, bacterial vaginosis, China

## Abstract

**Introduction:**

*Chlamydia trachomatis* is the etiological agent of the commonest sexually transmitted bacterial infection. This study aimed to examine the prevalence of genital chlamydia and associated risk factors in Chinese female outpatients with genital tract infections.

**Methods:**

A prospective, multicenter epidemiological study of genital chlamydia prevalence in 3008 patients with genital tract infections in 13 hospitals in 12 provinces of China was performed between May 2017 and November 2018. Vaginal secretion specimens were collected for the clinical diagnosis of vaginitis, whereas cervical secretion specimens were tested for *Chlamydia trachomatis* and *Neisseria gonorrhoeae*. All patients participated in a one-on-one cross-sectional questionnaire interview.

**Results:**

Totally 2,908 participants were included. The prevalence rates of chlamydia and gonococcal infections in women with genital tract infections were 6.33% (184/2908) and 0.01% (20/2908), respectively. Multivariate analysis showed high risk factors for chlamydia were premarital sex behavior, first sexual intercourse before the age of 20 and bacterial vaginosis.

**Discussion:**

Given that most chlamydia cases are asymptomatic and no vaccine is currently available, chlamydia prevention strategies should include behavioral interventions as well as early screening programs to identify and treat individuals with genital tract infections, especially those with the above identified risk factors.

## Introduction

Chlamydia is a commonly diagnosed sexually transmitted infection (STI) caused by *Chlamydia trachomatis* (*C. trachomatis*). In 2020, the World Health Organization (WHO) estimated 129 million individuals were infected by *C. trachomatis* worldwide; meanwhile, around 374 million new STI cases were detected, indicating more than 1 million STI cases acquired daily (1), which is likely an underestimate as chlamydia infections are often asymptomatic. In the United States, the Centers for Disease Control and Prevention (CDC) estimated almost $16 billion in direct medical costs alone, and the highest prevalence (61%) was detected among individuals aged 15–24 years (2). Most cases of chlamydia infection are generally asymptomatic. Multiple sequelae can result from chlamydia infection in women; especially if left untreated, the most serious sequelae may include pelvic inflammatory disease (PID), ectopic pregnancy, infertility and chronic pelvic pain, requiring high medical costs (3, 4). Over the past two decades, the rates of reported chlamydia cases have steadily increased in both men and women. During the COVID-19 pandemic, the temporary decreases observed in chlamydia infection rates from 2018 to 2021 in the United States were unlikely due to an actual reduction in new infections, as yearly reproductive health visits for young women also decreased during this period (3).

Genital tract infections can be classified anatomically and mainly involve vaginitis, cervicitis and PID (Table 1) (5). The pathogen targets endocervical and upper genital tract squamocolumnar epithelial cells in women, causing multiple urogenital infections such as cervicitis, acute salpingitis and PID; chlamydia is transmitted via direct contact during vaginal, anal or oral sex and may even be acquired by newborns during vaginal delivery. Moreover, chlamydia can coexist with other reproductive tract infections and STIs, including gonococcal infections, syphilis, human immunodeficiency virus infection (HIV), human Papillomavirus Infection and trichomoniasis (6). Therefore, the clinical management of urogenital chlamydia is focused on reducing the course of infection or inflammation (e.g., early diagnosis and treatment), preventing further sexual transmission and adverse reproductive health complications, and preventing *C. trachomatis* transmission to neonates during delivery (7).

**Table 1:** Classification of genital tract infections.

Infectious diseases	Anatomical site	Clinical syndrome
Vaginitis	Vagina	Trichomoniasis^a^	Vulvovaginal candidiasis^b^	Bacterial vaginosis^c^	Aerobic vaginitis^d^	Nonspecific vaginitis
Cervicitis	Cervix	Mucopurulent cervicitis	Cervical neoplasia
Pelvic inflammatory disease	Endometrium	Endometritis	Fallopian tubes	Salpingitis	Ovaries	Oophoritis
Others	Vulva	Vulvar vestibulitis	Vulva	Genital warts	Bartholin glands	Bartholinitis

^a^A type of vaginitis which caused by infection with the protozoan parasite *Trichomonas* vaginalis.

^b^A type of vaginitis which caused by infection with *Candida* species.

^c^Bacterial vaginosis refers to the relative lack of vaginal endogenous lactobacilli and synergistic anaerobic microbial growth in the vaginal epithelium.

^d^Refers to the relative lack of vaginal endogenous lactobacilli and synergistic aerobic microbial growth in the vaginal epithelium.

Chlamydia screening programs aim at reducing chlamydia and PID rates in women by early detection and treatment. The U.S. CDC therefore recommends annual screening for all sexually active women aged ≤25 years or older with high risk of infection, including those with new or multiple sexual partners (2). Meanwhile, other high-income countries, including the United Kingdom, Australia, Sweden, Denmark, and Norway, recommend annual chlamydia screening for sexually active young adults, especially women.

Genital chlamydia was included as a reportable STI in 105 national STI surveillance sites in China in 2008 (6). Despite the lack of official data in China, reports suggest 4.7–30% of women of reproductive age experience at least one chlamydia episode, with up to 70% of infections being asymptomatic (6, 8, 9); this rate ranges from 10 to 13% in pregnant women (10). Other studies revealed (8, 11, 12) chlamydia infection rate is even higher in women visiting sexually transmitted disease and gynecology clinics, reaching 10–20%. Considering the geographic variation of *C. trachomatis* infection, the severe situation of chlamydia and the lack of robust epidemiological studies, the current prospective, multicenter epidemiological study aimed to determine the epidemiological features and risk factors associated with chlamydia infection in gynecology clinics in China. Targeted screening is the most cost-effective strategy to reduce the prevalence of chlamydia (13). The current study provides guidance for nationwide chlamydia screening programs to reduce the burden of chlamydia in China.

## Materials and methods

### Patients

This multi-center, cross-sectional study was conducted at 13 hospitals in 12 provinces of China between May 2017 and November 2018. During this period, 6,125 women with gynecological complaints (e.g., vulvovaginal Itching, abnormal discharge, bleeding or abdominal pain) seeking sexual and reproductive health care were assessed. Among them, totally 3,008 women with genital tract infections were eligible for this study, and all of them were willing to participate face-to-face interview. All enrolled patients in the selected hospitals were required to participate in a questionnaire interview at the time of first visit after meeting the eligibility criteria and providing written informed consent. Inclusion criteria were: (1) with one or more types of genital tract infections, including vaginitis, cervicitis, pelvic inflammatory disease and so on; (2) ever engaged in sexual intercourse; (3) no antibiotic use in the last 2 weeks. Exclusion criteria were: (1) antimicrobial therapy during the previous 21 days; (2) previous history of hysterectomy; (3) menopause; (4) contraindications to the Papanicolaou test or cervical sampling.

### *Chlamydia trachomatis* questionnaire

A close-ended questionnaire was generated to assess potential risk factors associated with chlamydia. All enrolled patients in the selected hospitals were required to complete a face-to-face questionnaire interview at the time of enrollment after meeting the eligibility criteria and providing written informed consent. The questionnaire included five primary domains: (a) demographic data such as age, housing situation, monthly income, education level, marital status, and single parenthood; (b) personal hygienic behaviors, including the frequencies of vulva cleansing, vaginal douche, sanitary pads, and wearing cotton underwear; (c) sexual behaviors, including premarital sex, sexual orientation, age at first intercourse, frequency of sex, number of sex partners, experience of masturbation, experience of orgasm and use of a contraceptive device during sex; (d) history of gynecological diseases, including pregnancy, abortion and genital tract infections; (e) self-reported symptoms and signs such as vulvar pruritus, vulvar pain, increased vaginal discharge, vaginal odor, abdominal pain, lower urinary tract symptoms, postcoital bleeding, vaginal pH and vaginal cleanliness.

### Gynecological examination and clinical diagnosis of genital tract infections

Vaginal secretions were obtained and rolled onto glass slides with aseptic cotton swabs for microbiological tests. Cervical swab specimens were collected with the Cobas PCR Sample Kit (Roche Molecular Systems) following speculum examination.

Vaginitis was defined as inflammation or infection of the vagina, and had a broad differential types, including trichomoniasis, vulvovaginal candidiasis (VVC), bacterial vaginosis (BV), aerobic vaginitis (AV), and nonspecific vaginitis (Table 1) (14). The clinical diagnoses of trichomoniasis was based on wet mount for direct examination of *Trichomonas* in vaginal secretions, while the diagnosis of candidiasis was based on Gram staining for the detection of *Candida* hyphae, pseudohyphae or spores in vaginal secretions; the diagnosis of BV was performed as described by Nugent et al. (15) and scores valued 7–10 are interpreted as BV flora; the diagnosis of AV was performed as described by Donders et al. (16) and scores valued 3–10 accompanied by genital complaints are interpreted as AV (4, 14). The type of vaginitis that does not meet the criteria presented above but involved some of the features of vaginitis, was diagnosed as nonspecific vaginitis (17). Mixed vaginitis was defined as the simultaneous presence of at least two above types of vaginitis (18).

Cervicitis was clinically diagnosed according either or both major signs: (1) a visibly purulent or mucopurulent endocervical exudate, or (2) sustained endocervical bleeding easily induced by gentle contact of a cotton swab (4). PID was clinically diagnosed according one or more of the following three minimum clinical criteria present on pelvic examination: (1) cervical motion tenderness, (2) uterine tenderness, or (3) adnexal tenderness (4).

### *Chlamydia trachomatis* testing

Cervical specimens were transferred to tubes containing cervical cell preservation solution provided in the kit and stored at −80°C until analysis as described by the manufacturer. All samples were transported to Peking University First Hospital for the detection of *C. trachomatis*/*Neisseria gonorrhoeae* (*N. gonorrhoeae*) with a qualitative nucleic acid amplification test on the Cobas 4,800 system (Roche, Switzerland) as described by the manufacturer.

### Ethical approval of the study protocol

The study followed the Declaration of Helsinki and was approved by the Ethics Committee of Peking University First Hospital, Beijing, China (reference number: 2016[1156]). Written informed consent was provided by all eligible participants before enrolment. The participating institutions included Peking University First Hospital, Peking University Shenzhen Hospital, Beijing Tsinghua Changgung Hospital, The Second Affiliated Hospital of Chongqing Medical University, The First Affiliated Hospital of Guangxi Medical University, The Second Hospital of Hebei Medical University, Nanjing Drum Tower Hospital, Qinghai Red Cross Hospital, Qilu Hospital of Shandong University, The Second Hospital of Shanxi Medical University, Tianjin Medical University General Hospital, Women’s Hospital of Zhejiang University and The Third Xiangya Hospital of Central South University.

### Statistical analyses

IBM SPSS Statistics 23 (IBM Corp. Released 2016, IBM SPSS Statistics for Windows, Version 23, Armonk, NY; IBM Corp.) was used for statistical analysis. Data were reported as number (percentage) or odds ratio (OR) with the corresponding 95% confidence interval (CI). The chi-square test was performed to compare categorical variables between groups. Logistic regression analysis and raw ORs in univariate analysis were used to assess the associations of chlamydia with demographic and behavioral variables. *p* < 0.05 indicated statistical significance.

## Results

### Patient population

**Figure 1:**
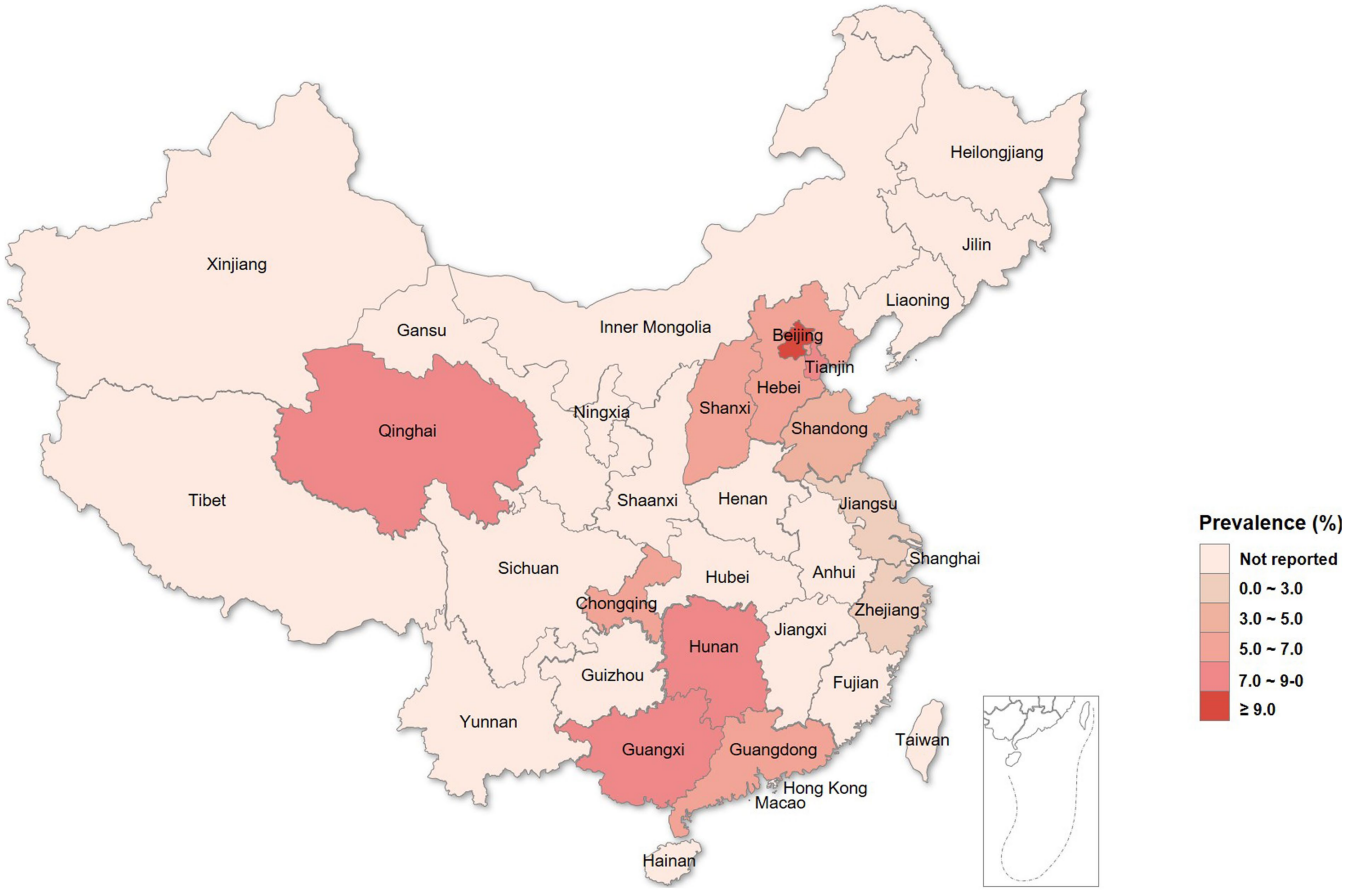
Prevalence of chlamydia in female outpatients with genital tract infections from different regions in China.

Totally 3,008 women were enrolled in this study, and 100 (3.3%) were excluded for poor sample quality or a lack of personal data. Finally, 2,908 (96.7%) evaluable patients were included in the analysis, including 2,222 women diagnosed with vaginitis, 393 with cervicitis, 131 with PID, and 271 with other genital tract infections (e.g., vulvitis and bartholinitis). The demographic and baseline characteristics of the patients are shown in Table 2. The prevalence rates of *C. trachomatis* and gonococcal infections in women with genital tract infections were 6.33% (184/2908) and 0.01% (20/2908), respectively; 6 cases were co-infected with *C. trachomatis* and *N. gonorrhoeae* (diagnosed with BV), nonspecific vaginitis, vulvovaginal candidiasis (VVC), cervicitis, BV + PID and cervicitis, respectively. As shown in Table 2, individuals with chlamydia were significantly younger than uninfected women (30.50 ± 7.087 yrs. vs. 34.98 ± 8.824 yrs., *p* < 0.001). Significant differences in *C. trachomatis* prevalence were found among regions in China, and women with genital tract infections in Beijing may have the highest burden in *C. trachomatis* prevalence, followed by Tianjin, Nanning, and Changsha (Table 2 and Figure 1).

**Table 2:** Characteristics of the study patients with *C. trachomatis* and *N. gonorrhoeae* infections.

	*C. trachomatis*			*N. gonorrhoeae*		
	Negative	Positive	*P*-value	Negative	Positive	*P*-value
Mean age, years	34.98 ± 8.824	30.50 ± 7.087	<0.001	34.69 ± 8.771	36.00 ± 11.549	0.618
Age at menarche, years	13.84 ± 1.629	13.97 ± 1.653	0.686	13.85 ± 1.633	13.50 ± 1.249	0.359
Parturition frequency	1.62 ± 1.568	1.25 ± 1.689	0.548	2.37 ± 1.11	2.37 ± 1.11	
Region, n (%)						
Beijing	410 (90.71%)	42 (9.29%)	0.024	446 (98.67%)	6 (1.33%)	0.610
Changsha	231 (92.77%)	18 (7.23%)		247 (99.20%)	2 (0.80%)	
Taiyuan	188 (94.47%)	11 (5.53%)		198 (99.5%)	1 (0.50%)	
Nanning	185 (92.50%)	15 (7.50%)		199 (99.5%)	1 (0.50%)	
Xining	158 (92.94%)	12 (7.06%)		168 (98.82%)	2 (1.18%)	
Nanjing	234 (98.32%)	4 (1.68%)		238 (100.0%)	0 (0.00%)	
Tianjin	185 (92.50%)	15 (7.50%)		200 (100.0%)	0 (0.00%)	
Shenzhen	364 (93.09%)	27 (6.90%)		387 (98.98%)	4 (1.02%)	
Hangzhou	165 (97.06%)	5 (2.94%)		170 (100.0%)	0 (0.00%)	
Shijiazhuang	233 (94.72%)	13 (5.28%)		245 (99.59%)	1 (0.41%)	
Jinan	190 (95.48%)	9 (4.52%)		198 (99.50%)	1 (0.50%)	
Chongqing	180 (93.26%)	13 (6.73%)		191 (98.96%)	2 (1.04%)	

To examine the role of the microecological environment of the female genital tract in chlamydia, we discussed the associations of genital tract infections.

with chlamydia. As shown in Table 3, BV prevalence was 34.27% (61/178) in *C. trachomatis* positive women, which was significantly higher than that of *C. trachomatis* negative women [27.43% (727/2,650)]. Chlamydia was associated with BV (OR = 1.379, 95%CI 1.000–1.901; *p* = 0.049). The prevalence of cervicitis was 30.00% (6/20) in *N. gonorrhoeae* positive women, which was significantly elevated than that of *N. gonorrhoeae* negative women [13.78% (387/2,808); Table 3]. *N. gonorrhoeae* infection was associated with cervicitis with an odds ratio of 2.681 (95% 1.024–7.019, *p* = 0.037). These results indicated tight associations of *C. trachomatis* and *N. gonorrhoeae* infections with genital tract infections.

**Table 3:** Prevalence rates of *C. trachomatis* and *N. gonorrhoeae* infections in 2828 patients with gynecological infectious diseases.

	n^a^	*C. trachomatis*, n (%)			*N. gonorrhoeae*, n (%)		
Negative		Positive^b^	*P*-value	Negative	Positive	*P*-value
Vaginitis	2,222	2079 (78.45%)	143 (80.34%)	0.553	2,205 (78.53%)	17 (85.00%)	0.482
TV	121	113 (4.26%)	8 (4.49%)	0.883	121 (4.31%)	0 (0.00%)	0.343
BV	788	727 (27.43%)	61 (34.27%)	0.049	779 (27.74%)	9 (45.00%)	0.086
VVC	715	675 (25.47%)	40 (22.47%)	0.373	712 (25.36%)	3 (15.00%)	0.288
AV	212	201 (7.58%)	11 (6.18%)	0.491	212 (7.55%)	0 (0.00%)	0.201
Nonspecific	765	715 (26.97%)	50 (28.09%)	0.745	760 (27.06%)	5 (25.00%)	0.837
Mixed vaginitis	312	290 (10.94%)	22 (12.36%)	0.559	312 (11.11%)	0 (0.00%)	0.114
Cervicitis	393	366 (13.81%)	27 (15.17%)	0.612	387 (13.78%)	6 (30.00%)	0.037
PID	131	124 (4.68%)	7 (3.93%)	0.646	129 (4.59%)	2 (10.00%)	0.252
Others	271	251 (9.43%)	20 (10.87%)	0.520	269 (9.52%)	2 (10.00%)	0.942

^a^Numbers did not always add up to the expected total because of 80 missing data.

^b^178 cases with *C. trachomatis* infection had data on reproductive tract infection status.

TV, Trichomonas vaginitis; BV, Bacterial vaginosis; VVC, Vulvovaginal candidiasis; AV, Aerobic vaginitis; PID, Pelvic inflammatory disease.

### Factors associated with chlamydia

Based on univariate analysis, risk variables associated with chlamydia in women with genital tract infections included unstable housing situation, unmarried/widowed/divorced marital status, premarital sex behavior, first sexual intercourse before the age of 20, having ≥3 sex partners, previous history of pregnancy, and previous history of abortion.

### Basic information

Our data demonstrated salient differences in individual *C. trachomatis* risks between women living in stable residences, including own houses or apartments, and those living in unstable situations (rooming houses, hotels or shelters). Individuals with a stable housing situation showed a 5.54% positivity rate, versus 8.40% in those with an unstable housing situation. Besides, the positivity rate of chlamydia in the unmarried/widowed/divorced group was 11.9%, which was significantly higher than 5.0% obtained in married women.

### Sexual behavior

In this study, another risk factor significantly associated with chlamydia was the sexual behavior. The prevalence of chlamydia in patients with the first sexual intercourse at an age below 20 years was significantly higher than that of patients having the first sexual intercourse at an age above 20 years (9.48% vs. 5.14%; *p* < 0.001). The prevalence of chlamydia in patients with 1 or 2 sexual partners over the lifetime was 10.05%, which was significantly lower than that of patients with 3 or more sexual partners (6.24%). The prevalence of chlamydia in patients with premarital sexual behavior was significantly higher than that of participants without premarital sexual behavior (7.76% vs. 3.81%; *p* < 0.001).

### Gynecological history

Among women with genital tract infections, the positivity rate of chlamydia in those without a previous pregnancy was 10.48%, which was significantly higher than that of participants who reported a past pregnancy (4.91%). A pregnancy history was inversely correlated with chlamydia risk (OR = 0.441, 95%CI 0.324–0.600; *p* < 0.001; Table 4). Similarly, the positivity rates of chlamydia were 7.64 and 5.21% in patients without and with a history of abortion, respectively.

**Table 4:** High-risk factors of *C. trachomatis* infection.

Variable		n^a^	*C. trachomatis* Positive n (%)	*P*-value
Basic information				
Age, years	≤25	404	31 (7.67%)	0.232	>25	2,503	153 (6.11%)	
Housing situation	Stable	2074	115 (5.54%)	0.005	Unstable	786	66 (8.40%)
Monthly income, RMB	<5,000	1,629	102 (6.26%)		0.768	≥5,000	1,023	67 (6.55%)
Education	High school or below	962	54 (5.61%)	0.245		Bachelor or higher	1900	128 (6.74%)
Marital status	Unmarried/Widowed/divorced	539	64 (11.87%)		<0.001	Married	2,320	116 (5.00%)
Single parenthood	No	2,687	167 (6.22%)	0.205		Yes	187	16 (8.56%)	
Personal hygienic behaviors				
Frequency of vulvar cleansing	Never	118	11 (9.32%)	0.524	Less than once a week	239	14 (5.86%)		2–6 times a week	474	32 (6.75%)		Daily	2057	125 (6.08%)	
Frequency of vaginal douche	Never	1,599	95 (5.94%)	0.771	Less than once a week	570	36 (6.32%)		2–6 times a week	306	22 (7.19%)		Daily	368	26 (7.07%)	
Use of sanitary pads	<5 days a month	2,374	140 (5.90%)	0.110	≥5 days a month	486	38 (7.82%)	
Cotton panties	No	386	17 (4.40%)	0.097	Yes	2,511	166 (6.61%)
Sexual behavior				
Premarital sex behavior	Yes	971	145 (7.76%)	<0.001	No	1869	37 (3.81%)	
Sexual orientation	Heterosexuality	2,731	173 (6.33%)	0.844	Homosexuality	49	4 (8.16%)		Bisexuality	1	1 (0.00%)	
Age at first intercourse, yrs	≤20	781	74 (9.48%)	<0.001	>20	2,103	108 (5.14%)	
Frequency of sex	≤ once a week	1,383	86 (6.22%)	0.404	2–3 times a week	1,268	85 (6.70%)		>4 times a week	172	7 (4.07%)	
Number of sex partners	1–2	2,386	149 (6.24%)	0.030	≥3	219	22 (10.05%)	
Experience of masturbation	No	2,480	151 (6.09%)	0.169	Yes	336	27 (8.04%)	
Experience of orgasm	No	597	46 (7.71%)	0.109	Yes	2,200	130 (5.91%)	
Using a contraceptive device during sex	No	1970	132 (6.70%)	0.197
	Yes	833	45 (5.40%)	
Gynecological history				
Pregnancy	No	735	77 (10.48%)	<0.001	Yes	2098	103 (4.91%)	
Abortion	No	1,296	99 (7.64%)	0.008	Yes	1,535	80 (5.21%)	
Genital tract infections	No	697	48 (6.89%)	0.505	Yes	2,153	133 (6.18%)	
Self-reported symptoms and signs				
Vulvar pruritus	No	1,594	102 (6.40%)	0.466	Yes	1,187	68 (5.73%)	
Vulvar pain	No	2,572	161 (6.26%)	0.257	Yes	209	9 (4.31%)	
Increased vaginal discharge	No	1,515	90 (5.94%)	0.674	Yes	1,265	80 (6.32%)	
Vaginal odor	No	1928	115 (5.96%)	0.613	Yes	851	55 (6.46%)	
Abdominal pain	No	2,422	153 (6.32%)	0.252	Yes	357	17 (4.76%)	
Lower urinary tract symptoms	No	2,617	159 (6.08%)	0.743	Yes	164	11 (6.71%)	
Postcoital bleeding	No	2,629	159 (6.05%)	0.523	Yes	150	11 (7.33%)	
Vaginal pH	Normal (3.8~4.4)	1,021	51 (5.00%)	0.044	Abnormal	1,312	92 (7.01%)	
Vaginal cleanliness^b^	Normal (I–II)	905	46 (5.08%)	0.034	Abnormal (III–IV)	1,584	115 (7.26%)	

^a^Numbers did not always add up to the total expected because of missing data.

^b^Vaginal cleanliness was used to judge inflammation status in wet preparations of vaginal swab samples, degree I-II representing normal vaginal microecological status while degree III-IV representing abnormal status (19).

RMB, Chinese Yuan Renminbi.

### Others

However, no significant associations were found of chlamydia with income, education level, single parenthood, personal hygienic behaviors, sexual orientation, frequency of sex, history of genital tract infections and self-reported symptoms and signs.

### Multivariate analysis

Significant variates in univariate analysis were further assessed by multivariate analysis, including age, housing situation, premarital sex behavior, age at first intercourse, number of sex partners, vaginal cleanliness, and BV. In the multivariate logistic model, factors remaining significantly associated with chlamydia in women were positive premarital sex behavior, first sexual intercourse before the age of 20 years and BV (Table 4).

## Discussion

This cross-sectional epidemiological study was performed to assess the epidemiology of chlamydia and to identify potential risk factors that could elevate the incidence of chlamydia in mainland China. We previously reported a *C. trachomatis* prevalence of 2.2% among married women in Beijing (20), versus 17.30% in female sex workers (21), and 10.1% in patients visiting gynecology and reproductive health clinics (12). This study showed that the prevalence of chlamydia has now increased by 3-fold in Chinese female outpatients with genital tract infections compared that married women. This change appears to be mostly affected by co-existing genital tract infections, especially vaginitis, suggesting *C. trachomatis* screening is particularly important in patients with vaginitis, and the vaginal microecology may play a key role in chlamydia. Screening for *C. trachomatis* is largely dependent on the available tests and accessible to the high-risk population (11); thus, in this research we assessed female outpatients with genital tract infections as the target population (Table 5).

**Table 5:** Odds ratios (ORs) in multivariate analysis.

Variable		*P*-value	OR	Upper limit	Lower limit
Age, years	≤25		1.000	1.000	1.000	>25	0.544	0.869	0.553	1.366
Housing situation	Stable		1.000	1.000	1.000	Unstable	0.134	1.313	0.919	1.876
Premarital sex behavior	Yes		1.000	1.000	1.000	No	0.005	1.903	1.218	2.972
Age at first intercourse, years	≤20		1.000	1.000	1.000	>20	0.024	0.665	0.467	0.948
Number of sex partners	1–2		1.000	1.000	1.000	≥3	0.471	0.827	0.492	1.388
Vaginal cleanliness	Normal (I–II)		1.000	1.000	1.000	Abnormal (III–IV)	0.185	1.288	0.886	1.874
BV	No		1.000	1.000	1.000	Yes	0.050	1.435	1.000	2.058

BV, bacterial vaginosis.

This study also indicated chlamydia is more prevalent in females with lower age at first intercourse. The tendency for chlamydia to concentrate geographically and its associations with social and neighborhood factors have long been established. Though the current case-based data do not include migrant status, we noted relatively high prevalence rates in the Beijing–Tianjin–Hebei urban agglomeration and southwestern coastal regions that are considered national migration destinations with intensive social-economic exchanges and frequent population movements, which may accelerate the spread of this sexually transmitted pathogen.

In this study, univariate analysis indicated that patients with unstable housing situation were disproportionately affected by the *C. trachomatis* epidemic. Unstable housing situation was associated with homelessness, poor physical health, mental illness and high risk of HIV (22). These findings indicate the importance of circumstances beyond individual behaviors for chlamydia risk, and environmental conditions and stable housing must be considered. Programs focusing on improving housing situation, might in turn exert indirect effects that may prove beneficial in preventing or reducing risky sexually transmitted behavior.

Multivariate analysis showed that premarital sex, younger age at the time of first sexual intercourse, and number of sex partners were associated with a higher risk of chlamydia, which may yet contribute to increasing the prevalence of STIs. Therefore, chlamydia control programs applying sex education strategies should focus on younger individuals, as well as other people with identified risk factors, including reducing early sex experience and the number of sex partners, to reduce the prevalence and economic burden of chlamydia. Furthermore, the prevalence of *C. trachomatis* was much higher in infertile patients compared with women with previous history of pregnancy or abortion. This result further supports the data demonstrating that chlamydia imposes a substantial reproductive burden in China.

The clinical condition of BV, in which the characteristic vaginal microbial communities *Lactobacillus* spp. are instead dominated by multiple anaerobes, is associated with an increased risk of *C. trachomatis* (23). Some of these anaerobes produce metabolites, e.g., biogenic amines and short chain fatty acids, which bolster chlamydia persistence (24). BV can therefore help identify individuals at high risk of chlamydia. Opportunistic screening for *C. trachomatis* among young sexually active adults has been recommended in outpatients with genital tract infections.

In terms of self-reported symptoms and signs, we found no symptoms or signs were significantly associated with the prevalence of chlamydia, indicating it is hard to detect *C. trachomatis* via healthcare seeking behaviors, and screening for the high-risk population is important.

This study had several limitations, and the results should be interpreted with caution. First, the study participants were not randomly recruited, and may not be an accurate representation of the target study population. Secondly, the history of genital tract infections was self-reported; over- or underreporting due to self-reporting bias may impact the association estimates.

In summary, this study provides novel insights into specific risk factors associated with chlamydia in 12 provinces of China. Given that most chlamydia infections are asymptomatic and no vaccine is currently available (25), the backbone of chlamydia prevention should include not only behavioral interventions but also early screening programs to identify and treat individuals with genital tract infections (26), detecting missed infections that could lead to reproductive complications, reducing the prevalence and economic burden of trachomatis infections.

## Data availability statement

The raw data supporting the conclusions of this article will be made available by the authors, without undue reservation.

## Ethics statement

The studies involving human participants were reviewed and approved by the Ethics Committee of Peking University First Hospital, Beijing, China. The patients/participants provided their written informed consent to participate in this study.

## Author contributions

ZL and QL: conception and design of the study. DZ, ZL, QL, SF, MH, YH, XH, HW, ZX, HX, FX, MX, XY, and JZ: investigation and data collection. DZ, ZL, and TL: data analysis. TL and DZ: drafting the manuscript. All authors commented on the draft and decision to submit.

## Conflict of interest

The authors declare that the research was conducted in the absence of any commercial or financial relationships that could be construed as a potential conflict of interest.

## Publisher’s note

All claims expressed in this article are solely those of the authors and do not necessarily represent those of their affiliated organizations, or those of the publisher, the editors and the reviewers. Any product that may be evaluated in this article, or claim that may be made by its manufacturer, is not guaranteed or endorsed by the publisher.

## Abbreviations

STI: sexually transmitted infection
*C. trachomatis*: *Chlamydia trachomatis*
WHO: World Health Organization
CDC: the Centers for Disease Control and Prevention
PID: pelvic inflammatory disease
HIV: human immunodeficiency virus infection
BV: bacterial vaginosis
*N. gonorrhoeae*: *Neisseria gonorrhoeae*
OR: odds ratio
CI: confidence interval
VVC: vulvovaginal candidiasis
